# A Note on Ledum

**Published:** 1888-05

**Authors:** 


A NOTE ON LEDUM may be serviceable in ascites associated with the gouty diathesis. A prominent symptom is constant chilliness, though at midnight there may come a sense of suffocation and heat, patient throwing off the bedclothes and becoming very restless. Ledum, patient is morose, discontented, much interested in the subject at hand.

